# Development and In Vitro Evaluation of an Innovative “Dietary Flavonoid Supplement” on Osteoarthritis Process

**DOI:** 10.1155/2017/7503240

**Published:** 2017-03-07

**Authors:** Maria Rosaria Lauro, Lucia Crascí, Francesca Sansone, Venera Cardile, Anna Maria Panico, Giovanni Puglisi

**Affiliations:** ^1^Department of Pharmacy, University of Salerno, Via Giovanni Paolo II, 84084 Fisciano, Italy; ^2^Department of Drug Science, University of Catania, Viale A. Doria, 95100 Catania, Italy; ^3^Department of Biomedical and Biotechnological Sciences, Section of Physiology, University of Catania, Via Santa Sofia 64, 95125 Catania, Italy

## Abstract

The aim of this study was to evaluate the antidegenerative effect in osteoarthritis damage of eriocitrin alone and eriocitrin formulated as innovative “dietary flavonoid supplement.” A complexation between eriocitrin and hydroxypropyl *β*-cyclodextrin by solubilization/freeze-drying method was performed. The complex in solution was evaluated by phase solubility studies and the optimal 1 : 2 flavanone/cyclodextrin molar ratio was selected. Hydroxypropyl *β*-cyclodextrin was able to complex eriocitrin as confirmed by UV-Vis absorption, DSC, and FTIR studies. The complex formed increased the eriocitrin water solubility (from 4.1 ± 0.2 g·L^−1^ to 11.0 ± 0.1 g·L^−1^) and dissolution rate (from 37.0% to 100%) in 30 min. The in vitro studies exhibit the notion that eriocitrin and its complex inhibit AGEs in a similar manner because hydroxypropyl *β*-cyclodextrin does not interfere with the flavanone intrinsic property. Instead, the presence of cyclodextrin improves eriocitrin antioxidant stability maintaining a high fluorescence value until 8 hours with respect to the pure materials. Moreover, hydroxypropyl *β*-cyclodextrin showed moderate GAGs restoration acting synergistically with the complexed compound to maintain the structural chondrocytes integrity. The results point out that ERT/HP-betaCD complex possesses technological and biological characteristics able to obtain an easily soluble nutraceutical product, which reduces the degenerative and oxidative damage which occurs in osteoarthritis, and improve the patient compliance.

## 1. Introduction

Osteoarthritis (OA) is a progressive degenerative joint disease with a high impact on quality of life. During the OA process, a number of aging-related changes in joint tissue structure and function are involved. In the literature, it is reported that oxidative stress is extremely increased in OA and it is responsible for the etiopathogenesis of the disease [1]. Oxidative damage may be caused by chondrocyte senescence and cartilage aging [2, 3], with a loss of extracellular matrix (ECM) turnover and an increase of matrix metalloproteinases (MMPs) and cytokines production [4].

Moreover, the Advanced Glycation End products (AGEs) accumulation in many tissues, including the joints, is related to oxidative stress and contributes to the development of OA [5]. In fact, elevated AGEs levels lead to augmented MMPs production by chondrocytes, especially collagenases, which in turn induce degradation of cartilage and release of proteoglycans (PG) fragments [6].

On these bases, the reduction of oxidative stress, AGEs accumulation, and PG release may represent a good therapeutic approach in the prevention of the cartilage destruction in OA.

The latest research suggests that “conscious nutrition” from fruits and vegetables can be able to protect bone mass in time [7]. Nutraceuticals and dietary supplements derived from herbs play an important role in inflammation and joint destruction in OA [8]. In fact, since among risk factors there is poor intake of natural anti-inflammatory compounds such as some flavonoids, antioxidants, and essential fatty acids, the treatment plan for OA includes also medical nutrition with a blend of natural ingredients from phytochemical food [9]. A market formulation of a mixture of two polyphenols, baicalein and catechins, respectively, active in inhibiting cyclooxygenase and lipoxygenases in in vitro and in vivo animal models, is just used for the treatment of chronic OA [10]. Moreover, in the literature, it is reported that many* Citrus* flavonoids inhibit the cartilage degradation process, via MMPs inhibition [11], with a reduction of glycosaminoglycans (GAGs) release and restoration of nitric oxide (NO) levels [12].

**Figure 1:**
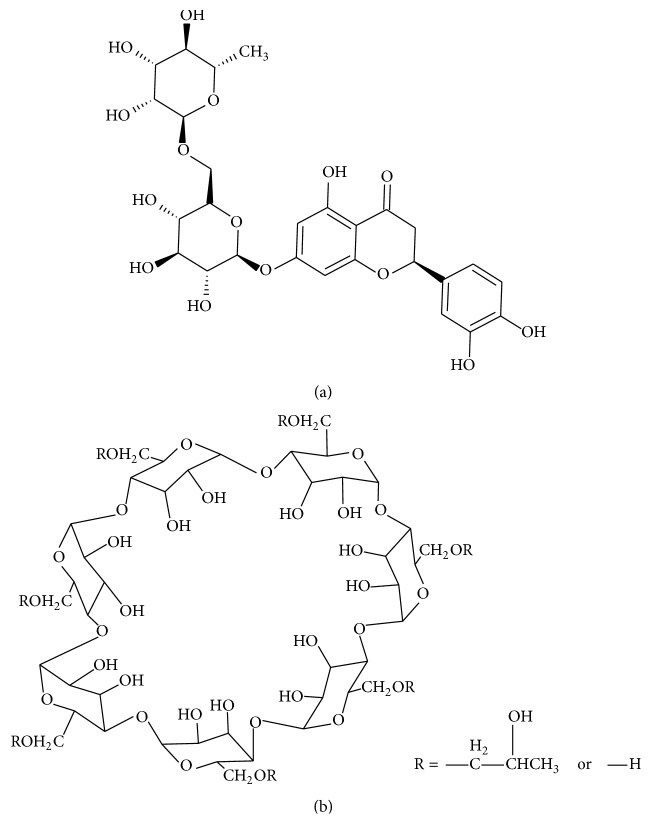
Chemical structures of eriocitrin ((a), ERT) and 2-hydroxypropyl *β*-cyclodextrin ((b), HP-betaCD).

The purpose of this study was to investigate the antidegenerative effect of eriocitrin (ERT) (Figure 1(a)), a flavanone glycoside abundantly present in lemon and lime fruits [13]. We developed an innovative easily handled powder formulation with improvement of ERT technological characteristic to produce a new “dietary flavonoid supplement” able to protect from OA damage and to enhance patient compliance. The limiting of flavonoids use in therapy is due to their low water solubility, a characteristic which leads to a very low dissolution rate and in vivo irregular absorption when administered as an oral solid dosage form [14, 15]. For this reason, hydroxypropyl *β*-cyclodextrin (HP-betaCD) (Figure 1(b)) was employed to develop ERT/hydroxypropyl *β*-cyclodextrin inclusion complex as easily soluble and stable powders of ERT dietary supplement. HP-betaCD is a soluble derivative of *β*-cyclodextrin and was selected because in the literature it is reported that it forms inclusion complexes with flavonoids and enhances their aqueous water solubility and dissolution rate [16–18]. Moreover, HP-betaCD appears to be useful in many diseases correlated to an increase of GAGs [19, 20], with PG also involved in OA.

In order to establish the ERT : HP-betaCD molar ratio, phase solubility studies have been applied and in vitro dissolution tests analyses have been also performed on ERT and its inclusion complex.

To evaluate the ability of flavanone alone and flavanone formulated to protect from OA damage, we assayed the antioxidant efficacy (ORAC assay) and antiglycation activity. The capability to prevent glycosaminoglycans (GAGs) release in human articular chondrocytes stimulated with interleukin-1*β* (IL-1*β*) cellular model of OA [21, 22] was also evaluated.

## 2. Materials and Methods

### 2.1. Chemicals

Eriocitrin (ERT) was supplied by Sigma-Aldrich (Milan, Italy). Hydroxypropyl *β*-cyclodextrin (HP-betaCD) was purchased from Cyclolab (Cyclolab Cyclodextrin Research & Development Laboratory, Ltd., Hungary, medium molecular weight: 8500 g mol^−1^). All other chemicals used were of reagent grade.

### 2.2. Phase Solubility Studies

*Eriocitrin Solubility*. The solubility of ERT was previously determined in water (*s* = 4.1 g·L^−1^) as follows: an excess amount of raw ERT was introduced into a flask containing 50 mL of solvent; according to the “shake flask method” [23], the sample was shaken for 3 days and then stored at room temperature (25°C) [24]. After 3 days, the amount of dissolved flavonoid was determined by HPLC and UV method of the supernatant solution previously filtered.

*UV-Vis Method*. UV-Vis assay was performed according to ICH [Q2(R1)] guideline and validated statistically using % Relative Standard Deviation (% RSD). Active concentration in the supernatant was evaluated by measuring absorbance at *λ*max ERT of 284 nm in 1 mm cell (UV-Vis 1601 Shimadzu Europa, Duisburg, Germany). The concentration of the dissolved substances in the sample is calculated by Lambert-Beer Law according to USP 37:

(1) $$\begin{array}{c} A={E^{1\%}}_{1\;cm}\times c\times l, \end{array}$$

where *E*^1%^_1 cm_ is the absorbance of 1 g/100 mL (1% w/v) solution in 1 cm cell, *c* is the concentration of the solution (g/100 mL), and *l* is the path length of the cell which the sample is held in.

To validate the method, the active concentration was calculated using also the standard calibration curve.* Linearity.* The proportionality between absorbance and concentration was verified at room temperature at five concentration levels in the range of 25.0 to 250.0 mg·L^−1^ (*y* = 32.769*x* − 0.235, *R*^2^ = 0.999, where *y* is the absorbance and *x* is the concentration used). Reference standard solutions were prepared in triplicate and 5 mL of each solution was analyzed in triplicate and the results are expressed as % average value ±  % RSD.

*HPLC Method*. ERT concentration was also evaluated by an HPLC apparatus (Varian Prostar mod. 230, Varian, Milan, Italy) equipped with a 20 *μ*L Rheodyne 7125 injection valve (Rheodyne, Cotati, CA, USA) autosampler Varian mod. D10 (Varian, Milan, Italy) and a UV-Vis detector set at *λ* 284 and Software Galaxie. Chromatographic analyses were performed on a LiChrospher® 100 C18RP column (particle size 5 *μ*m; 250 × 4 mm ID; Merck, Darmstadt, Germany), equipped with a 5 *μ*m LiChrospher 100 C18RP guard column (4 × 4 mm ID; Merck, Darmstadt, Germany) and eluted isocratically at room temperature. The mobile phase consisted of a 50 : 50 (v/v) mixture of methanol/water; the flow rate was set at 1.0 mL/min.* Linearity.* Reference standard solutions were prepared in triplicate at five concentration levels (0.1–1.0 *μ*g·mL^−1^). 20 *μ*L of each solution was analyzed in triplicate. The standard curve was analyzed using linear least-squares regression equation derived from the peak areas (*y* = 1611.4*x* − 0.32, *R*^2^ = 0.999) where *y* is the peak area and *x* is the concentration used.* Specificity.* The peak associated with ERT was identified by retention time and confirmed by coinjection, as well as by UV (UV-Vis 1601 Shimadzu Europa, Duisburg, Germany).

*Isotherm Solubility and Stability Constant*. The solubility phase diagrams of the inclusion complex were determined by Higuchi and Connors's method [25]. An excess amount of ERT (1.0 × 10^−3^ mol) was suspended in 100 mL of water and then different amounts of HP-betaCD in the molar ratios 1 : 0.25, 1 : 0.5, 1 : 1, 1 : 1.5, 1 : 2, and 1 : 3 (ERT/HP-betaCD) were added. Six samples for each molar ratio were prepared, shaken, stored at 25°C, and then checked at 15, 30, 45, 60, 75, and 90 min after centrifugation for 5 min at 3000 rpm. The supernatants were analyzed in UV apparatus in 1 cm cell at *λ* 284 nm and, after 1 hour, equilibrium was reached.

Each experiment was carried out in triplicate (RSD < 3%).

### 2.3. Complex Preparation and Characterization

*Preparation of the Solid Complex*. The ERT/HP-betaCD solid complex was prepared with the solubilization/freeze-drying method [26] as follows: 2 × 10^−4^ mol of ERT was suspended in 100 mL of water and then was added with 4 × 10^−4^ mol of HP-betaCD. The sample was vortexed for 60 sec, stored for 1 hour at −4.0°C, and lyophilized for 24 h to obtain the clathrate ERT/HP-betaCD.

*Absorption Spectra*. The absorption spectra of ERT/HP-betaCD complex (2 : 1 molar ratio) and raw materials were taken with a Shimadzu UV-Vis spectrometer model UV-1601. The molar concentrations examined were 2 × 10^−2^ mol for ERT and 4 × 10^−2^ mol for HP-betaCD.

*Drug Content and Inclusion Efficiency (IE)*. The drug content and IE in ERT/HP-betaCD were assessed by both UV and HPLC.

*UV Method.* Samples (2 mg) of each complex were dissolved in 3 mL of water and vortexed for 60 sec. The drug content was determined spectrophotometrically (UV-Vis 1601, Shimadzu, MA, USA) at *λ* 284 nm (1 mm cell; SpectraComp 602, Advanced Products Srl, Milan, Italy). Each analysis was made in triplicate and the results were expressed as average value. Values were superimposable to those obtained by HPLC analyses.

*HPLC Method*. Three samples (2 mg) of ERT, ERT/HP-betaCD physical mixture (ERT/HP-betaCD mix), and ERT/HP-betaCD complex were dissolved in 3 mL H_2_O, vortexed for 60 sec, and centrifuged at 3000 rpm for 5 min. The concentration was determined in the supernatant solutions using the same chromatographic conditions described in the precedent section. Each analysis was performed in triplicate and the results, expressed as average value, are reported in Table 1.

The inclusion efficiency (IE) was calculated from the ratio of actual drug content (ADC) to theoretical drug content (TDC) in a freeze-dried complex (Table 1).

*Differential Scanning Calorimetry (DSC)*. Raw materials, ERT/HP-betaCD mix, and ERT/HP-betaCD complex were analyzed by differential scanning calorimetry on an indium calibrated Mettler Toledo DSC STARe (Mettler Toledo, OH, USA). Thermograms were recorded by placing accurately given quantities (1-2 mg weighed with a microbalance MTS Mettler Toledo, OH, USA) of each sample in a 40 *μ*L aluminium pan which was sealed and pierced. The sample was scanned (10°C/min) between 25 and 350°C [27]. Melting temperature (*T*_*m*_) and heat of fusion (Δ*H*_*m*_) were measured.

*Fourier Transform Infrared Spectroscopy (FTIR)*. Fourier transform infrared spectra were obtained using a Jasco FT-300 (Tokyo, Japan) Fourier transform IR (FTIR) spectrometer. Samples of complex CD/IDB and of raw materials were analyzed as KBr discs in the spectral region 400–4000 cm^−1^.

### 2.4. In Vitro Dissolution Studies

The sample of complex corresponding to about 2.4 mg of ERT (sink conditions) was analyzed spectrophotometrically at *λ* 284 nm in water.

In vitro dissolution/release test of ERT/HP-betaCD complex was carried out in 1000 mL of water under sink conditions (corresponding to about 2 g/L of ERT) using a SOTAX AT Smart Apparatus (Basel, CH) in line with a spectrophotometer at 284 nm (UV-Vis spectrometer Lambda 25, PerkinElmer Instruments, MA, USA) and USP 37 dissolution test apparatus n.2: paddle, 100 rpm at 37°C. All the dissolution/release tests were performed in triplicate; only the mean values are reported in the graph.

ERT alone and ERT/HP-betaCD physical mixture (ERT/HP-betaCD mix) were used as control.

### 2.5. Antioxidant Efficiency (ORAC Assay)

A modified oxygen radical absorbance capacity (ORAC) assay was used to determine the antioxidant qualitative activity of ERT, HP-betaCD, and ERT/HP-betaCD complex [11]. The tested compounds (25 *μ*L) were placed in 96-well tissue culture plates. 150 *μ*L of fluorescein (FL) (10 nM) was used as the probe to assess the antioxidant activity. 25 *μ*L of the water-soluble azo-compound 2,2′-azobis(2-amidinopropane) dihydrochloride (AAPH, 100 mM) was used as a radical initiator to generate free radicals at a constant rate. A positive control (FL solution containing AAPH), a negative control (FL solution containing no AAPH), and all the samples tested (1 mg·mL^−1^) were run simultaneously. A timer was started upon introduction of the free radical generator and the plate was stored in the dark at 37°C. At each specified time point, the fluorescence of the solution was measured (excitation 492 nm, emission 535 nm) using a Wallac 1420 Victor 3 96-well plate reader (PerkinElmer, USA) fluorimeter and plotted as a function of time with Origin®7 (OriginLab Corporation, Northampton, USA). The *y*-axis graph was split as follows: 5000 to 6000 RFU.

### 2.6. Antiglycation Activity

The inhibition of fluorescence produced by AGEs formation through Maillard reaction [28] was evaluated. The protein model bovine serum albumin (BSA) (10 mg/mL) was incubated with D-fructose (0.5 M) in a phosphate buffer (50 mM, pH 7.4) (NaN_3_ 0.02%) to obtain positive controls. BSA alone was the negative control corresponding to no fluorescence AGEs formation. Aminoguanidine (AMG) (400 *μ*g/mL) was used as a reference compound for its AGEs inhibition property [29]. The final glycated BSA solutions (300 *μ*L) alone and with ERT, HPBCD, or ERT/HPBCD complex (400 *μ*g/mL) were incubated at 37°C in 96-well microtiter plates closed with their silicon lids for 7 days. The AGEs fluorescence measurement (*λ*exc 370 nm; *λ*em 440 nm) was performed using a Victor Wallac 1420 Multilabel Counters fluorimeter (PerkinElmer, USA). The results are reported in relative fluorescence units (RFU) and the percentage of inhibition with respect to the positive control (BSA with fructose) and are calculated from the following equation:

(2) %  of  inhibition=1−RFU  samplenmRFU  Positive  controlnm×100.

### 2.7. Biological Cellular Assay

#### 2.7.1. Human Articular Chondrocytes Culture

Normal human articular cartilage was obtained at replacement surgery from healthy patients (30–40 years old) and with femoral neck accidental fractures. Informed consent was previously obtained. The isolation procedure was conducted under antiseptic conditions. The cartilage was cut into small fragments and carefully washed using Dulbecco's Modified Eagle's Medium (DMEM) containing NaHCO_3_, 25 mM Hepes, 1 mM sodium pyruvate, 50 mg/mL gentamycin, 100 U·mL^−1^ penicillin, 100 mg·mL^−1^ streptomycin, and 2.5 mg·mL^−1^ amphotericin B. Chondrocytes were isolated through three sequential passages of enzymatic digestion of the extracellular matrix: incubation with 0.1% hyaluronidase type III (1 mg·mL^−1^ for 100 mg of cartilage), for 30 min at 37°C; incubation with 0.5% pronase type XIV (5 mg·mL^−1^ for 100 mg of cartilage), for 60 min at 37°C; and, finally, incubation with 0.2% collagenase type IA (2 mg·mL^−1^ for 100 mg of cartilage), for 45 min at 37°C. The obtained cellular suspension was filtered (filters from 100 and 70 mm) to eliminate the residues of the digestion, cellular aggregates. Freshly isolated chondrocytes were seeded into monolayer culture at a cell density of 2 · 10^5^ cells and cultured in 1 mL of DMEM supplemented with 10% foetal bovine serum (FBS) at 37°C in 5% CO_2_/95% air [30]. Confluent chondrocytes of primary culture were plated and treated as follows: untreated control, IL-1*β* at 10 ng/mL as a positive control, and the tested compounds (ERT, HPBCD, and ERT/HPBCD complex), dissolved in DMSO and appropriately diluted in Dulbecco's Modified Eagle's Medium (DMEM) at two final concentrations (10 *μ*g·mL^−1^ and 100 *μ*g·mL^−1^). After 48 and 72 hours, the supernatants of chondrocytes culture were collected for different assays.

#### 2.7.2. Cell Viability Assay

The cytotoxic effect of the experimental substances was evaluated by a cell viability test based on the cleavage of 3-(4,5-dimethylthiazol-2-yl)-2,5-diphenyltetrazolium bromide (MTT) by mitochondrial dehydrogenases of metabolically active cells [31].

#### 2.7.3. Determination of GAGs

The level of GAGs, an index of cartilage damage, was measured by spectrophotometry with a solution of 1,9-dimethylmethylene blue at *k* = 535 nm. The amount of glycosaminoglycans was calculated from a standard curve (100–500 *μ*g/mL) obtained for shark chondroitin sulphate C, derived from shark cartilage [32].

## 3. Results and Discussion

ERT was formulated in the presence of HP-betaCD polymer to obtain a complex active in osteoarthritis disease and with improvement of its aqueous solubility and dissolution rate.

### 3.1. Phase Solubility Studies

**Figure 2:**
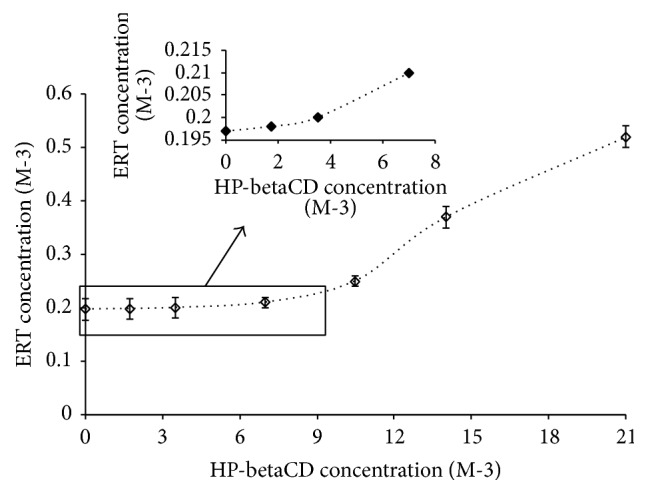
Solubility phase diagram of ERT in the presence of HP-betaCD.

The poor water ERT solubility (4.1 ± 0.2 g·L^−1^) at room temperature was notably affected by the presence of HP-betaCD. In fact, ERT/HP-betaCD complex showed water solubility of 11.0 ± 0.1 g/L. According to Connors and Higuchi [25], the presence of HP-betaCD gave an A-type phase solubility profile typical of soluble cyclodextrin with a positive deviation from linearity (Ap-type, Figure 2). This indicates that the complex is first order compared with the substrate but is second or higher order compared with the ligand. The most common stoichiometry associated with the Ap profile is the 1 : 2 active/HP-betaCD molar ratio complex.

For this reason, conventionally, we assumed a 1 : 2 ERT/HP-betaCD molar ratio to obtain an inclusion complex (ERT/HPBCD complex) by solubilization/lyophilization method.

### 3.2. UV-Vis Absorption Study

**Figure 3:**
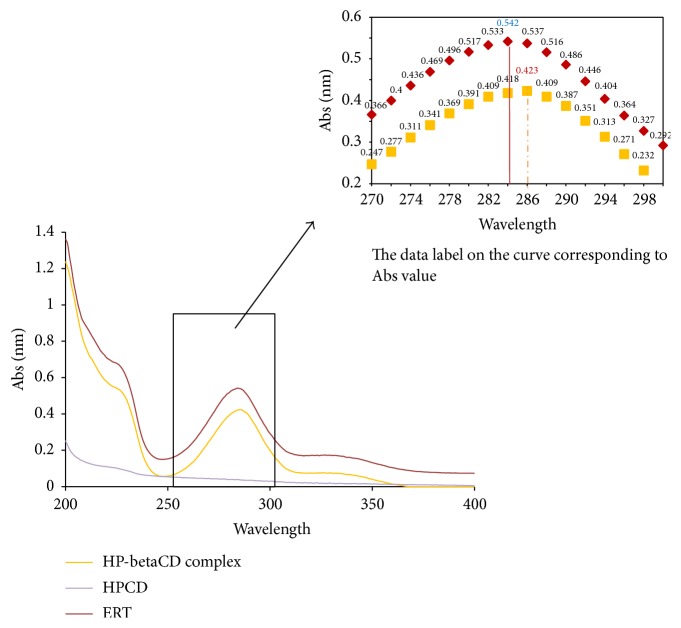
UV spectra analyses of ERT/HP-betaCD complex in comparison with ERT and HP-betaCD pure materials.

The interactions of ERT and HP-betaCD in the aqueous solution can be examined by comparing the UV-Vis spectra of ERT with those of the inclusion compound (Figure 3). It is possible that both inclusion and noninclusion forms coexist in the solution, so we performed this test in water in which the complexes were highly soluble compared with ERT. ERT spectrum is according to *n* → *π* and *π* → *π*^*∗*^ associated with electronic transition of the carbonyl chromophore group alpha to the aromatic ring [33]. The intense maximum peak is at 284 nm (called band II) with a shoulder at 327 nm (called band I). Only in ERT/HP-betaCD complex was a slight modification both of the absorption maximum and of the wavelength values observed. Also, a modification of the absorption of band I was noted (from 327 nm to 330). According to literature data for other flavonoids analogue of ERT [34], the measurements were carried out taking into consideration band II of ERT. The addition of HP-betaCD led to a hypsochromic shift of 2 nm (from 284 nm to 286 nm) and a bathochromic effect (k band decrease in intensity). These results were probably due to the inclusion of ERT in HP-betaCD cavity suggesting an ERT/HP-betaCD complex formation with weak bonds [35].

### 3.3. Drug Content and Inclusion Efficiency (IE)

The actual ERT content values determined by both UV and HPLC analyses were in perfect agreement. The percentage of ERT in the samples ranged from 99.8 ± 0.1 to 100.1 ± 0.2 (*n* = 3), indicating that it was uniformly distributed (Table 1).

### 3.4. DSC and FTIR Analyses

**Figure 4:**
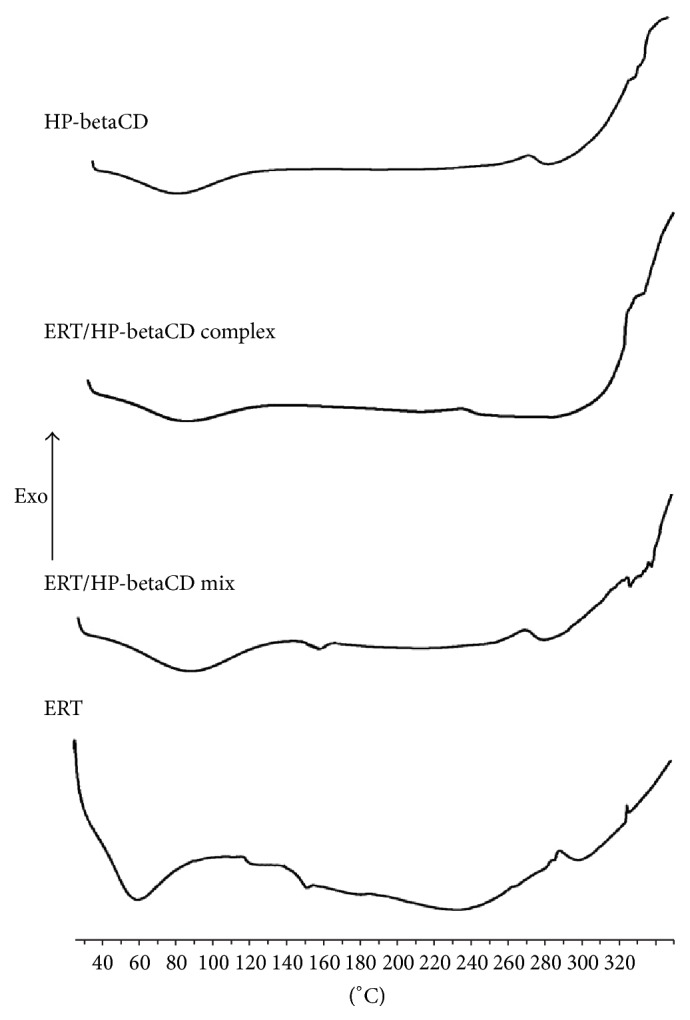
DSC of ERT and HP-betaCD alone, physical mixture (ERT/HP-betaCD mix), and ERT/HP-betaCD complex.

The DSC method reveals some information on host-guest solid state interactions. The occurrence of the complexation is usually evidenced by the decreased intensities or disappearance of the drug melting temperature [36]. ERT (Figure 4) showed a broad endothermic event between 35 and 90°C due to moisture loss. The “melting point” at 152°C was detected near a phase transition peak (135°C). As in other flavonoids, this was probably due to a molecular rearrangement of ERT polymorph in a plastic substance [37]. Peaks occurring over 250°C were due to the ERT decomposition. HP-betaCD and ERT/HP-betaCD mixture (ERT/HP-betaCD mix) exhibit an endothermic event in the range of 40–110°C, because of the loss of water. Moreover, in ERT/HP-betaCD mix ERT lower melting peak intensity may be due to the HP-betaCD lower fusion temperature that amorphized ERT. In the freeze-drying ERT/HP-betaCD system, ERT signal disappears definitively confirming the complex formation.

**Figure 5:**
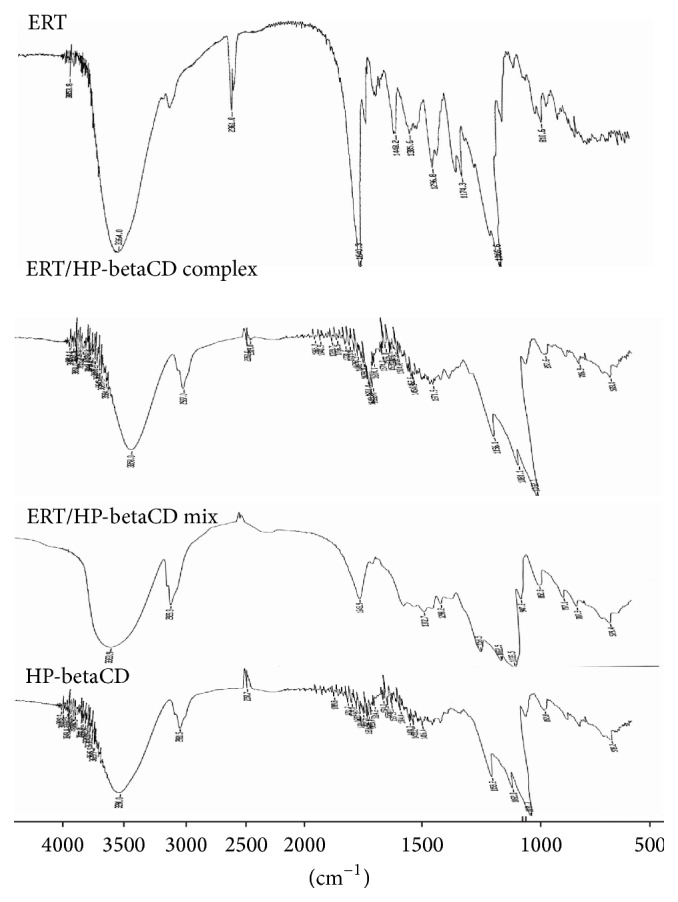
FTIR spectra (from 4000 to 500 cm^−1^) of ERT and HP-betaCD alone, physical mixture (ERT/HP-betaCD mix), and ERT/HP-betaCD complex.

The complexation process of ERT with the cyclodextrin has been confirmed by the FTIR spectroscopy (Figure 5). FTIR spectra of ERT/HP-betaCD complex show differences in the peak patterns with respect to raw materials. FTIR spectrum of ERT showed a band at 2925.2 cm^−1^ as a result of the CH-aliphatic vibration. The bands at 1643.4, 1622.1, and 1531.4 cm^−1^ correspond to that of the aromatic bond –C=C. The absorption bands in 1000–700 cm^−1^ interval are specific for the pulsation vibrations in glucopyranosyl unit and for the deformation vibrations of C-H bonds [34]. The HP-betaCD signals at 3390 cm^−1^, 2931.5 cm^−1^, 1159 cm^−1^, and 1082 cm^−1^ correspond to symmetrical, stretching, antisymmetric *ν* [OH], *ν* [CH_2_], and *ν* [CC], and bending *ν* [OH] phenomena, respectively [36]. The characteristic signals of both components of ERT/HP-betaCD mix are evident and indicate weaker or no interaction between ERT and HP-betaCD when physically mixed. Any new peaks were found in ERT/HP-betaCD complex spectra; HP-betaCD signals shift at lower fields confirming a physical interaction due to the presence of weak hydrogen bonds. Instead, both did not show bands typical for intensity and position. In particular, the ERT signal at 1640.3 and 1622 cm^−1^ disappears, while the signals shift (1600–1550 cm^−1^, C=C valence vibrations in the benzene rings; 950–750 cm^−1^, interval specific for the pulsation vibrations in glucopyranosyl unit and for the deformation vibrations of C-H bonds) and their intensity strongly decreases. These results suggest the inclusion of the ERT in the cavity of cyclodextrin and the formation of hydrogen bond between the flavanol and HP-betaCD.

### 3.5. In Vitro Dissolution/Release Test

**Figure 6:**
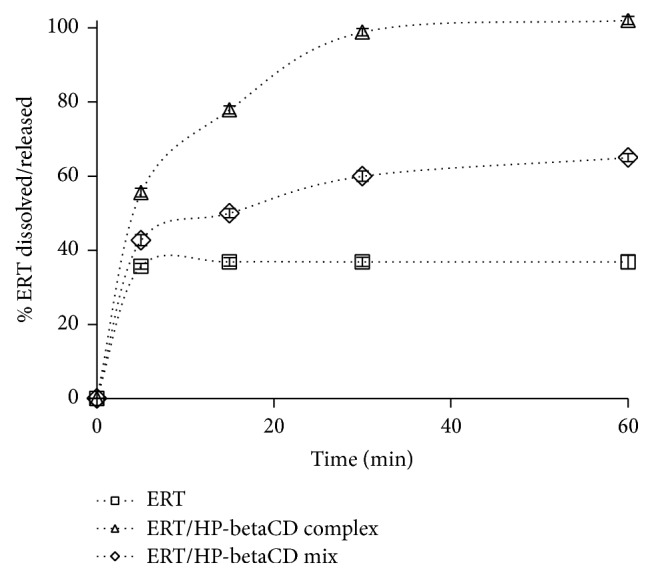
Dissolution/release profile of ERT/HP-betaCD complex, in comparison with physical mixture (ERT/HP-betaCD mix) and ERT pure material dissolution profiles in water.

The dissolution/release profiles of ERT from ERT/HP-betaCD complex in comparison with dissolution profiles of ERT/HP-betaCD mix and neat ERT in water are reported in Figure 6. All the results showed a standard deviation < 5%.

All the loaded dose (100.0%) of ERT was released in 30 min from ERT/HP-betaCD, while about 65.0% of the flavanone dissolved from ERT/HP-betaCD mix and only 37.0% of ERT alone dissolved at the same time. These results may be explained by an increase of the ERT water interaction due to the presence of soluble HP-betaCD polymer that was able to complex ERT and to improve drug wettability and water solubility. The technological features of the water-soluble powders developed can increase patients compliance reducing their discomfort to swallow solid dosage forms.

### 3.6. Antioxidant Efficiency

To verify the ability of ERT/HP-betaCD formulation to maintain or prolong the antioxidant capability of active included compound, we used the ORAC assay.

**Figure 7:**
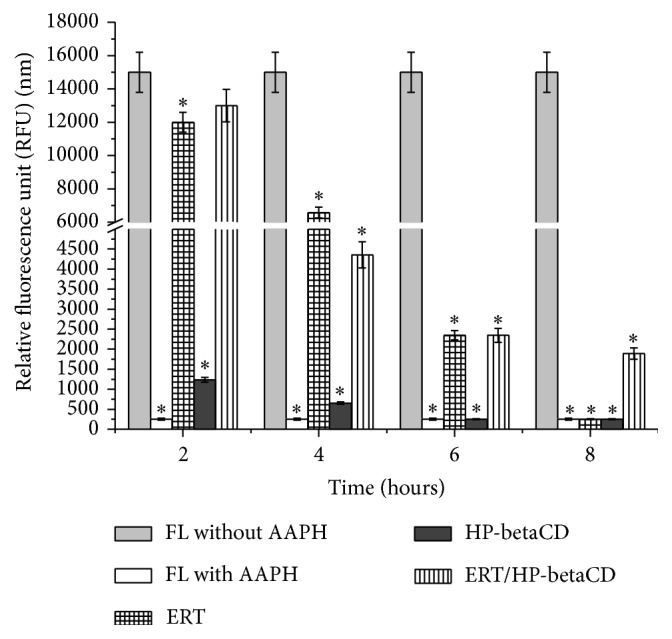
Antioxidant efficiency of ERT, HP-betaCD, and ERT/HP-betaCD complex. Data represent the mean of three independent experiments ± SD. ^*∗*^*p* < 0.05 compared with FL without AAPH.

Figure 7 reports the fluorescence value (RFU) in a function of time (hours) of all tested compounds. Both ERT and ERT/HP-betaCD complex possess a good capability to delay the reduction of FL fluorescence. Particularly, while HP-betaCD and ERT as pure materials lose their effect after 2 and 6 hours, respectively, ERT/HP-betaCD complex maintains a high fluorescence value (1980 nm) until 8 hours. This trend indicates that the formulation improves the ERT antioxidant stability, confirming its high scavenger radical effect.

### 3.7. Antiglycation Activity

In the literature, it is reported that oxidative/nitrosative stress and inflammation, which occur in OA disease [38], induce tissue AGEs accumulation that causes property modification of ECM components [39, 40] with consequent cartilage degradation [41].

**Figure 8:**
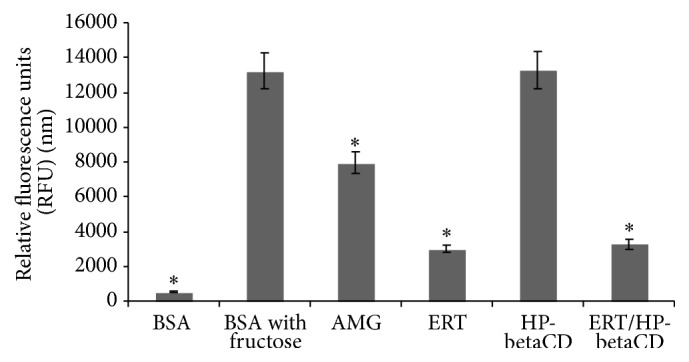
Antiglycation effect of ERT, HP-betaCD, and ERT/HP-betaCD complex. In comparison with AMG assay standard control. Results are means ± SD at ^*∗*^*p* < 0.05 compared with positive control (BSA with fructose).

On this basis, we evaluated the inhibitory effect of ERT and ERT/HP-betaCD complex on AGEs formation (Figure 8). Both samples reduce the max fluorescent value of positive control (BSA with fructose), compared with HP-betaCD alone that exhibits the same fluorescence value. This indicates that the cyclodextrin used does not interfere with the intrinsic property of ERT. In fact, ERT alone or formulated showed a good capability to inhibit the AGEs formation with 77.3% and 75.3% of inhibition (fluorescence value of 3006 nm and 3269 nm), respectively, maintaining a fluorescence value higher than reference assay standard AMG (40%).

### 3.8. Cellular Assay

IL-1*β* is a proinflammatory cytokine involved in the activation of different cellular OA mediators, as nitric oxide (NO), prostaglandin E_2_ (PGE_2_), and MMPs, causing degradation of structural collagen macromolecules, like GAGs [12]. Indeed, the chondrocytes stimulation with IL-1*β* (10 ng·mL^−1^) was used as a cellular model of OA to test the ability of compounds to protect the cartilage degradation process.

#### 3.8.1. MTT Assay

The MTT assay was performed to evaluate the cell viability after treatment with the tested compounds. The results showed that treatment for 72 hours reduces the ability of chondrocytes to metabolize tetrazolium at both used concentrations (10 *μ*g·mL^−1^ and 100 *μ*g·mL^−1^). Instead, treatment with 10 *μ*g·mL^−1^ for 48 hours did not interfere with the cells viability (data not shown). So, this dosage may be used to evaluate the GAGs effect of compounds.

#### 3.8.2. Determination of GAGs Levels

The cartilage degradation process in OA is due to cross-linking between AGEs, formed from inflammatory/oxidative stress, and long-lived proteins of ECM, contributing to a loss of ECM components such as GAGs levels, which represent an index of cellular damage [39].

**Figure 9:**
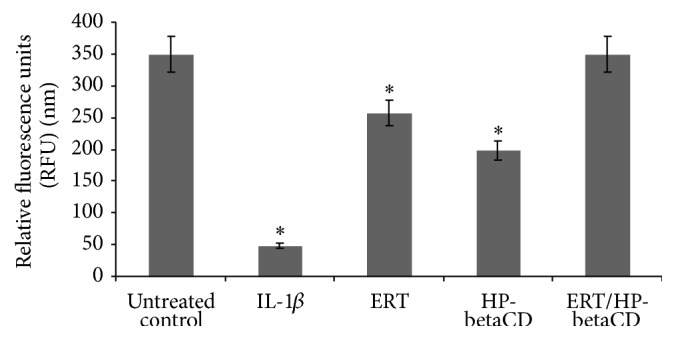
Determination of GAGs levels (*μ*g·mL^−1^) (means ± SD) in the culture medium of articular chondrocytes, stimulated with 10 ng·mL^−1^ of IL-1*β*, 48 hours after the addition of the tested compounds at 10 *μ*g/mL. ^*∗*^Significantly different from IL-1*β* treated samples (*p* < 0.05).

Figure 9 reports the GAGs levels, expressed in *μ*g/mL, on the supernatant of chondrocytes after treatment with IL-1*β* (10 ng·mL^−1^) and at the presence of tested compounds (10 *μ*g·mL^−1^). IL-1*β*, responsible for the altered cellular balance and ECM synthesis, induces a loss of GAGs levels (47.7 *μ*g·mL^−1^), which are significantly elevated (350 *μ*g·mL^−1^) in untreated control. Both ERT and ERT/HP-betaCD restore the GAGs levels, protecting the chondrocytes from the proinflammatory cytokine action. In particular, the GAGs levels of ERT/HP-betaCD (349 *μ*g·mL^−1^) are superimposable with that of untreated control, showing the structural integrity of chondrocytes. This is due partially to the effect of HPBCD acting synergistically with the complexed compound. In fact, it shows moderate GAGs restoration (198 *μ*g·mL^−1^) for its capability to form hydrogen bonds with polysaccharides, as GAGs [19]. From this result, the potential role of HP-betaCD as a therapeutic agent also emerges for the prevention and treatment of a group of metabolic disorders in which abnormal accumulations of GAGs occur.

## 4. Conclusions

Inflammatory/oxidative stress plays a central role in OA development. In fact, during cartilage degradation, an increase of proinflammatory cytokines, free radicals, metalloproteinases, and also AGEs accumulation occurs, which induce the release of ECM components as GAGs. In recent years,* Citrus* flavonoids were considered good agents to prevent OA damage [11]. On the bases of the renaissance of the plant derivatives and the consumer interest in novel healthy food ingredients, we developed and tested an innovative ERT/HP-betaCD inclusion complex (1 : 2 molar ratio) as dietary flavonoid supplement to be employed in OA. The results exhibit the ERT activity on oxidative/inflammatory and cartilage degenerative factors. In fact, the examined flavonoid was able to inhibit AGEs formation and restore GAGs. These ERT intrinsic activities improved when ERT is complexed with HP-betaCD, a soluble beta-cyclodextrin able to also enhance ERT solubility, wettability, and dissolution rate. Moreover, ERT/HP-betaCD complex improved ERT antioxidant effect and restored GAGs. This behavior can lead to increased in vivo ERT release and to the improvement of its bioavailability and activity. In addition, according to the literature [19, 20], an intrinsic activity of HP-betaCD on GAGs restoration also emerges, indicating its potential function in the treatment and prevention of many diseases associated with the imbalance of GAGs.

This approach might be suitable to obtain an extemporaneous solution of ERT active in OA damage and with an increase of patient compliance.

## Figures and Tables

**Table 1:** Percentage of theoretical drug content (TDC%), UV, and HPLC recovery of actual drug content (ADC), inclusion efficiency (IE%) of ERT in physical mixture (ERT/HP-betaCD mix) and in ERT/HP-betaCD inclusion complex (HP-betaCD/ERT complex), and amount of active ERT (ERT amount, %) in pure ERT and in the mixture.

Sample	TDC%	ADC UV% recovery	ADC HPLC% recovery	IE	ERT amount%
ERT	100	100.1 ± 0.2	100.2 ± 0.1	—	100.1 ± 0.2
ERT/HP-betaCD mix	100	99.8 ± 0.1	99.9 ± 0.1	—	99.8 ± 0.1
ERT/HP-betaCD complex	100	100.1 ± 0.1	100.1 ± 0.1	100.1 ± 0.1	

## References

[1] Altindag O., Erel O., Aksoy N., Selek S., Celik H., Karaoglanoglu M. (2007). Increased oxidative stress and its relation with collagen metabolism in knee osteoarthritis. *Rheumatology International*.

[2] Yudoh K., Nguyen V. T., Nakamura H., Hongo-Masuko K., Kato T., Nishioka K. (2005). Potential involvement of oxidative stress in cartilage senescence and development of osteoarthritis: oxidative stress induces chondrocyte telomere instability and downregulation of chondrocyte function. *Arthritis Research & Therapy*.

[3] Martin J. A., Brown T. D., Heiner A. D., Buckwalter J. A. (2004). Chondrocyte senescence, joint loading and osteoarthritis. *Clinical Orthopaedics and Related Research*.

[4] Loeser R. F. (2009). Aging and osteoarthritis: the role of chondrocyte senescence and aging changes in the cartilage matrix. *Osteoarthritis and Cartilage*.

[5] DeGroot J., Verzijl N., Wenting-Van Wijk M. J. G. (2004). Accumulation of advanced glycation end products as a molecular mechanism for aging as a risk factor in osteoarthritis. *Arthritis and Rheumatism*.

[6] Crascì L., Lauro M. R., Puglisi G., Panico A. (2016). Natural antioxidant polyphenols on inflammation management: anti-glycation activity vs metalloproteinases inhibition. *Critical Reviews in Food Science and Nutrition*.

[7] Macdonald H. M., New S. A., Golden M. H. N., Campbell M. K., Reid D. M. (2004). Nutritional associations with bone loss during the menopausal transition: evidence of a beneficial effect of calcium, alcohol, and fruit and vegetable nutrients and of a detrimental effect of fatty acids. *The American Journal of Clinical Nutrition*.

[8] Deal C. L., Moskowitz R. W. (1999). Nutraceuticals as therapeutic agents in osteoarthritis: the role of glucosamine, chondroitin sulfate, and collagen hydrolysate. *Rheumatic Disease Clinics of North America*.

[9] Akhtar N., Haqqi T. M. (2012). Current nutraceuticals in the management of osteoarthritis: a review. *Therapeutic Advances in Musculoskeletal Disease*.

[10] Patient Information Managing Osteoarthritis Pain, http://www.limbrel.com 19998991

[11] Lauro M. R., Crasci L., Carbone C., Aquino R. P., Panico A. M., Puglisi G. (2015). Encapsulation of a citrus by-product extract: development, characterization and stability studies of a nutraceutical with antioxidant and metalloproteinases inhibitory activity. *LWT—Food Science and Technology*.

[12] Crascì L., Panico A. (2013). Protective effects of many citrus flavonoids on cartilage degradation process. *Journal of Biomedical Nanotechnology*.

[13] Miyake Y., Shimoi K., Kumazawa S., Yamamoto K., Kinae N., Osawa T. (2000). Identification and antioxidant activity of flavonoid metabolites in plasma and urine of Eriocitrin-treated rats. *Journal of Agricultural and Food Chemistry*.

[14] Tommasini S., Raneri D., Ficarra R., Calabrò M. L., Stancanelli R., Ficarra P. (2004). Improvement in solubility and dissolution rate of flavonoids by complexation with *β*-cyclodextrin. *Journal of Pharmaceutical and Biomedical Analysis*.

[15] Picerno P., Sansone F., Mencherini T. (2011). *Citrus bergamia* juice: phytochemical and technological studies. *Natural Product Communications*.

[16] Kim H., Kim H.-W., Jung S. (2008). Aqueous solubility enhancement of some flavones by complexation with cyclodextrins. *Bulletin of the Korean Chemical Society*.

[17] Wen J., Liu B., Yuan E., Ma Y., Zhu Y. (2010). Preparation and physicochemical properties of the complex of naringenin with hydroxypropyl-*β*-cyclodextrin. *Molecules*.

[18] Yao Y., Xie Y., Hong C., Li G., Shen H., Ji G. (2014). Development of a myricetin/hydroxypropyl-*β*-cyclodextrin inclusion complex: preparation, characterization, and evaluation. *Carbohydrate Polymers*.

[19] Fumić B., Končić M. Z., Jug M. (2017). Therapeutic potential of hydroxypropyl-*β*-cyclodextrin-based extract of medicago sativa in the treatment of mucopolysaccharidoses. *Planta Medica*.

[20] Ren B., Jiang B., Hu R. (2016). HP-*β*-cyclodextrin as an inhibitor of amyloid-*β* aggregation and toxicity. *Physical Chemistry Chemical Physics*.

[21] Panico A., Cardile V., Santagati N. A., Messina R. (2009). Antioxidant and protective effects of Sumac Leaves on chondrocytes. *Journal of Medicinal Plants Research*.

[22] Vicini P., Crascì L., Incerti M., Ronsisvalle S., Cardile V., Panico A. M. (2011). Benzisothiazolyliminothiazolidin-4-ones with chondroprotective properties: searching for potent and selective inhibitors of MMP-13. *ChemMedChem*.

[23] Florence A. T., Attwood D. (2016). Physicochemical principles of pharmacy. *Manufacture, Formulation and Clinical Use*.

[24] Sansone F., Picerno P., Mencherini T. (2013). Enhanced technological and permeation properties of a microencapsulated soy isoflavones extract. *Journal of Food Engineering*.

[25] Connors K. A., Higuchi T. (1965). Phase solubility techniques. *Advances in Analytical Chemistry Instrumentation*.

[26] Sansone F., Mencherini T., Picerno P. (2014). Microencapsulation by spray drying of *Lannea microcarpa* extract: technological characteristics and antioxidant activity. *Journal of Pharmacy and Pharmacognosy Research*.

[27] Lauro M. R., Carbone C., Auditore R. (2013). A new inclusion complex of amlodipine besylate and soluble *β*-cyclodextrin polymer: preparation, characterization and dissolution profile. *Journal of Inclusion Phenomena and Macrocyclic Chemistry*.

[28] Derbré S., Gatto J., Pelleray A., Coulon L., Séraphin D., Richomme P. (2010). Automating a 96-well microtiter plate assay for identification of AGEs inhibitors or inducers: application to the screening of a small natural compounds library. *Analytical and Bioanalytical Chemistry*.

[29] Edelstein D., Brownlee M. (1992). Mechanistic studies of advanced glycosylation end product inhibition by aminoguanidine. *Diabetes*.

[30] Panico A., MacCari R., Cardile V., Crascí L., Ronsisvalle S., Ottanà R. (2013). 5-Arylidene-4-thiazolidinone derivatives active as antidegenerative agents on human chondrocyte cultures. *Medicinal Chemistry*.

[31] Graziano A. C. E., Parenti R., Avola R., Cardile V. (2016). Krabbe disease: involvement of connexin43 in the apoptotic effects of sphingolipid psychosine on mouse oligodendrocyte precursors. *Apoptosis*.

[32] Farndale R. W., Sayers C. A., Barrett A. J. (1982). A direct spectrophotometric microassay for sulfated glycosaminoglycans in cartilage cultures. *Connective Tissue Research*.

[33] Cavrini V., Andrisano V. (2013). Tecniche combinate. *Principi di Analisi Farmaceutica*.

[34] Corciova A., Ciobanu C., Poiata A. (2014). Inclusion complexes of hesperidin with hydroxypropyl-*β* -cyclodextrin. Physico-chemical characterization and biological assessment. *Digest Journal of Nanomaterials and Biostructures*.

[35] Ammar H. O., Salama H. A., Ghorab M., Mahmoud A. A. (2007). Inclusion complexation of glimepiride in dimethyl-*β*-cyclodextrin. *Asian Journal of Pharmaceutical Sciences*.

[36] Sambasevam K. P., Mohamad S., Sarih N. M., Ismail N. A. (2013). Synthesis and characterization of the inclusion complex of *β*-cyclodextrin and azomethine. *International Journal of Molecular Sciences*.

[37] Lauro M. R., De Simone F., Sansone F., Iannelli P., Aquino R. P. (2007). Preparations and release characteristics of naringin and naringenin gastro-resistant microparticles by spray-drying. *Journal of Drug Delivery Science and Technology*.

[38] Ziskoven C., Jäger M., Kircher J. (2011). Physiology and pathophysiology of nitrosative and oxidative stress in osteoarthritic joint destruction. *Canadian Journal of Physiology and Pharmacology*.

[39] Kurtz A., Oh S.-J. (2012). Age related changes of the extracellular matrix and stem cell maintenance. *Preventive Medicine*.

[40] Korpos E., Wu C., Sorokin L. (2009). Multiple roles of the extracellular matrix in inflammation. *Current Pharmaceutical Design*.

[41] Sophia Fox A. J., Bedi A., Rodeo S. A. (2009). The basic science of articular cartilage: structure, composition, and function. *Sports Health*.

